# Expired Patents

**Published:** 1907-12

**Authors:** 


### EXPIRED PATENTS.

November, 11, 1907.

440128. Dental Engine, W. G. A. Bonwill, Philadelaphia, Pa.
440131. Artificial Tooth, R. Brewster, London, England.
440509. Teeth Separator, F. Sawhill, Hastings, Neb.

The above lists of patents are furnished by Davis & Davis, Solicitors of American and Foreign Patents, Washington, D. C. and St. Paul Building, New York City.

